# Unusual pattern of herpetic optic neuropathy: a case report and literature review of the pathophysiology of herpetic uveitis

**DOI:** 10.1186/s12348-023-00335-4

**Published:** 2023-03-21

**Authors:** Rehab Sabry Helal, Rami Abu Sbeit, Zamzam Mohammed Al-Baker

**Affiliations:** Ophthalmology Department, Hamad Medical Corporation, Doha, Qatar

**Keywords:** Herpetic optic neuropathy, Pathophysiology of viral uveitis, Acute retinal necrosis, Non-necrotizing herpetic retinopathy.

## Abstract

Herpetic uveitis is a relatively common type of intraocular inflammation with a broad spectrum of manifestations ranging from mild anterior uveitis to rapidly progressing vision threatening necrotizing retinitis. Posterior herpetic uveitis presents with different clinical patterns within a spectrum depending presumably on the immune status of the patient. Systemic steroid use for viral uveitis without prior antiviral coverage is inappropriate and can lead to dramatic sequelae. Here, we report an unusual case of herpetic optic neuropathy in the contra lateral eye of a patient with acute retinal necrosis after improper use of oral steroids.

## Background

Human herpes viruses cause intraocular inflammation ranging from mild anterior uveitis to full blown necrotizing retinitis. Varicella zoster virus (VZV), Herpes Simplex Virus (HSV-1 and HSV-2) and Cytomegalovirus (CMV) are the usual culprits. Necrotizing herpetic retinitis is a rare form of herpetic uveitis and has 3 main clinical patterns: progressive outer retinal necrosis (PORN) and CMV retinitis in the immunocompromised, and acute retinal necrosis (ARN) in patients with presumably competent immune system. Since it was first described by Urayama et al. [1] in 1971, ARN was believed to be a unilateral disease consisting of acute unilateral panuveitis associated with vitritis, retinal periarteritis progressing to diffuse necrotizing retinitis and rhegmatogenous retinal detachment. Seven years later, the acronym BARN was used by Young and Bird to describe patients with bilateral ARN [2]. After Culbertson et al. [3] described its herpetic etiology in 1982, multiple herpes viruses have been implicated, VZV, HSV-1, HSV-2 being the most common, with reported cases confirming CMV and Epstein-Barr virus (EBV) [4, 5]. Bilateral ARN occurs in up to 70% of untreated patients, whereas those treated with intravenous acyclovir showed a significant decrease in risk of fellow eye involvement down to 13% [6]. Contralateral involvement usually occurs within few months but may occur years later [6].

PORN was described by Forster et al. [7] 1990 in HIV-positive patients with CD4 + T-cell counts less than 100/mm3. It resembles ARN with the following distinguishing features: (1) involvement of the outer retina; (2) the absence of any significant inflammation in the vitreous or aqueous humour; and (3) the absence of involvement of the retinal vasculature.

The umbrella term “Non-necrotizing herpetic retinopathies” was first used by Bodaghi et al. [8] 2003 to refer to five patients with non-necrotizing herpetic posterior uveitis consisting mainly of vasculitis, papillitis or vitritis.

Atypical “slow-type ARN'' has also been reported and the term was coined by Wensing et al. [9] in 2011. These newly described patterns tend to have better visual prognosis than the classic ARN.

## Case presentation

A 43-year-old male patient of south Asian descent presented to our eye casualty complaining of right eye pain and acute drop of vision over three days and left eye pain and progressive blurring of vision over the previous two weeks. He had initially presented ten days earlier to a general ophthalmologist elsewhere with left eye redness, pain and mild blurring of vision for several days and no complaints in the right eye, when his ophthalmologist diagnosed the condition as left eye iridocyclitis, and he prescribed topical prednisolone drops and oral prednisolone 30 mg for 5 days.

The patient was not known to have any systemic disease. He denied previous similar episodes, high risk sexual behavior or exposure to tuberculosis. Review of systems was unremarkable, including a negative history of any symptoms related to autoimmune diseases.

**Fig. 1:**
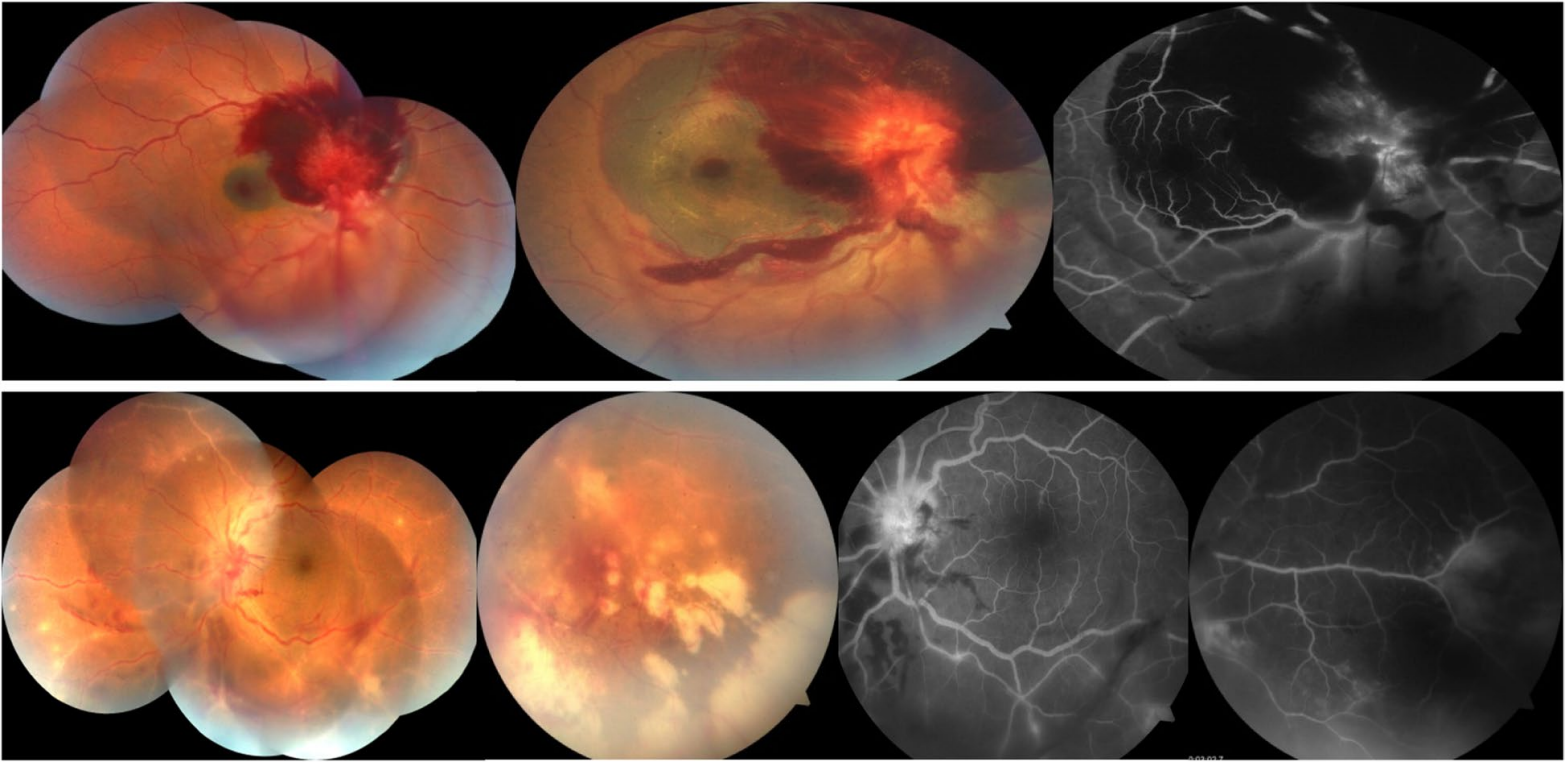
Clinical picture and FFA on presentation: upper column; Right eye montage and posterior pole colour fundus photos showing severe optic disc edema with multilayered retinal hemorrhages (subretinal, intraretinal and minimal localised inferior vitreous hemorrhage), with clear ocular media and normal retinal periphery. Mid venous phase Fundus fluorescein angiography of the same eye showing swollen leaky optic disc with extensive blockage of the choroidal filling by the multilayered retinal hemorrhages. Lower column; Left eye montage and peripheral colour fundus photos showing extensive retinal vasculitis with peripheral confluent patches of retinitis and satellite retinitis lesions. Fundus Fluorescein angiography of the same eye showing staining and leakage of retinal arteries and optic disc with staining of the peripheral areas of retinitis. Localized blockage of the dye caused by the peripapillary retinal hemorrhages

At his presentation in the emergency room, his vital signs and systemic examination were normal. The right eye’s (OD) best corrected visual acuity (BCVA) was 20/400 with normal intraocular pressure (IOP), occasional cells in anterior chamber (AC), localized inferior vitreous hemorrhage, severe swollen optic disc with multilayered peripapillary and subfoveal hemorrhages. The left eye (OS) had 20/100 BCVA, normal IOP, + 1 AC cells with medium size keratic precipitates (KP’s), mild vitreous reaction, hyperemic optic disc along with peripapillary splinter hemorrhages, diffuse extensive vasculitis with few hemorrhages and confluent well-defined patches of necrotic retina spread in a circumferential manner (Fig. 1).

The patient was referred the next day to the uveitis clinic for opinion and he was diagnosed as right eye acute retinal necrosis and left eye differential diagnosis was unusual pattern of optic neuropathy, hemorrhagic papilledema or Terson syndrome.

Fundus fluorescence angiography (Fig. 1) of the right eye showed a well-defined hypofluorescent area corresponding to the multilayered retinal hemorrhages and late papillary staining and leakage. The left eye showed vascular staining with diffuse disc leakage and staining of active retinitis patches.

Initial blood works showed normal complete blood count, kidney function, electrolytes, liver function, ESR and CRP.

Despite the left eye clear clinical picture of acute retinal necrosis the simultaneous right eye findings posed a diagnostic dilemma, in alignment with left eye findings.

The patient was admitted and started on intravenous acyclovir 10 mg/kg every 8h, diagnostic anterior chamber tap was performed for left eye only (as it showed significant inflammatory reaction promising more chances for diagnostic yield) and detailed uveitis work up was done. The aqueous humor sample was tested by PCR for HSV-1, HSV-2, VZV, CMV and EBV, and it came positive for VZV. Further workup was negative for infectious causes including HIV and syphilis. Patient PPD reading was 21 mm, QuantiFERON-TB gold test was positive and chest x-ray was normal. He was started on anti-TB treatment as latent TB. Brain MRI/MRA was done and it revealed normal brain study with no cerebral vasculitis and no evidence of any intracranial pathology.

Beside IV acyclovir, aspirin 100 mg was prescribed. Intravitreal ganciclovir injection, (2 mg/0.1 ml) was repeated every 3 days (total of 3 injections for each eye). He showed a good response on that treatment and no new retinal lesions developed. The retinal changes showed good regression. After one week of IV acyclovir, oral prednisolone was initiated at 80 mg daily.

After 15 days of hospital stay, the patient elected to travel to his home country. On discharge the BCVA remained 20/400 in the right eye and improved to 20/80 in the left eye, intraocular pressure was normal and the anterior segments of both eyes were quiet.The right optic disc and peripapillary and sub-foveal hemorrhages showed some improvement as well. Left eye retina was flat with around 50% healing of the retinitis patches.

The patient was advised to continue on oral valacyclovir 3000 mg daily and to taper oral prednisolone weekly after consulting a uveitis specialist in his home country promptly upon his arrival.

**Fig. 2:**
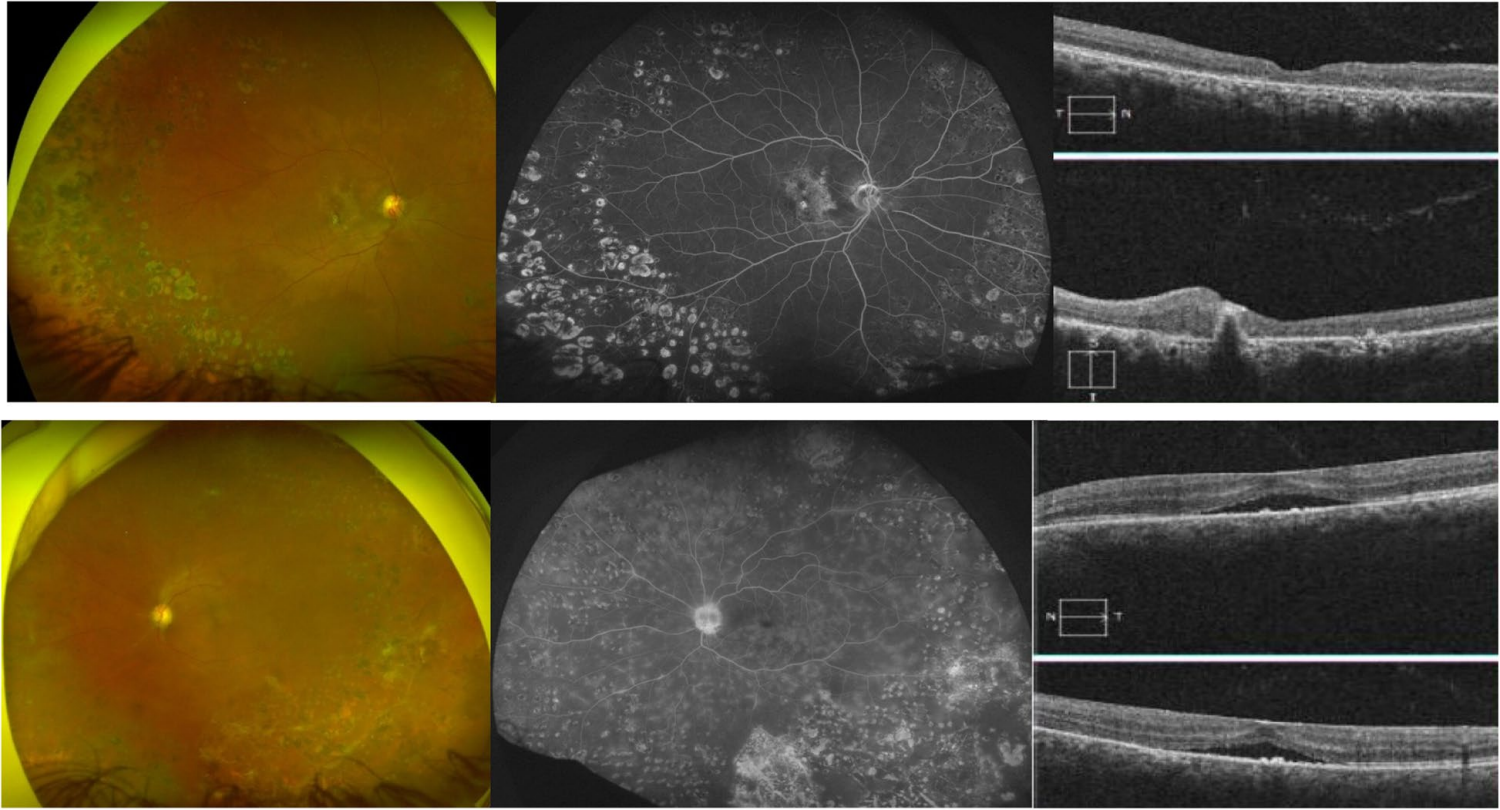
Last follow up Fundus photo, FFA and OCT). Upper column ( right eye): wide field fundus photo showing subtle temporal disc pallor with macular scarring and sheathed vessels more evident at the papillo-macular bundle and upper arcades. 360 degrees laser marks evident at the periphery. Wide field mid- transit FFA showing only staining of the laser and macular scars.OCT macula showing disorganized retinal layers with scarring involving mainly the outer retina and the retinal pigment epithelium (RPE) and focal RPE detachment. Lower column (left eye): wide field colored fundus photo showing more evident disc pallor, diffuse arterial sheathing and peripheral retinal scarring. Wide field FFA imaging showing diffuse significant capillary leakage with staining of the optic disc, peripheral retinal scars and laser marks. OCT macula: showing epiretinal membrane with flattening of the foveal pit, sub-macular fluid and RPE scarring

Patient returned back to our facility for follow-up after 18 months. He reported that he continued on oral valacyclovir for 6 months, and he was followed by an ophthalmologist in his home country, his condition remained inactive since then and he received laser treatment for both eyes. His examination showed, BCVA of 20/400 OD and 20/200 OS with normal IOP, quiet anterior segment both eyes. Left fundus showed moderate disc pallor, epiretinal membrane with absent foveal reflex, widespread significant arterial sheathing and peripheral retinal scars representing healed areas of previous retinal necrosis, his right fundus showed less obvious disc pallor and less significant sheathing of arterial vessels, the macula showed significant scarring with pigmentary changes. 360 degrees bilateral peripheral laser marks were evident both eyes (Fig. 2).

FFA was done and showed left eye diffuse capillary leakage but the right eye did not show any evidence of activity.

The capillary leakage in the left eye is representing a subclinical activity indicating the resumption of antiviral treatment.The patient was advised to resume his oral valacyclovir and referred to retina service for possible pars plana vitrectomy and epiretinal membrane peeling for the left eye with guarded visual prognosis.

## Discussion

Acute retinal necrosis used to be a clinical diagnosis based on the criteria defined by the Executive Committee of the American Uveitis Society in 1994: at least one area of peripheral retinal necrosis with circumferential spread, anterior chamber and vitreous inflammation, occlusive vasculopathy and rapid disease progression in the absence of therapeutic intervention [10]. After the molecular methods such as the polymerase chain reaction (PCR) became more available for clinician use, Takase et. al [11] in 2014 introduced new diagnostic criteria for ARN based on the ocular findings, clinical course, and virologic testing of intraocular fluids, delineating 2 levels of diagnosis: ‘‘virus-confirmed ARN’’ and ‘‘virus-unconfirmed ARN’’.

ARN occurs in seemingly immunocompetent patients, though functional abnormalities in cellular immunity have been described in ARN patients [12, 13], ARN cases have been described in HIV positive patients with limited immunosuppression retaining sufficient function to mount an inflammatory response [14] and PORN pattern was recently reported in immunocompetent patients [15].

Posterior herpetic uveitis in apparently healthy individuals takes several forms [8, 9, 15–17], Table 1. These forms may represent a continuum on the spectrum of viral retinopathies. The patterns other than PORN, classic ARN, and multifocal posterior necrotizing retinitis tend to have better visual prognosis [18]. The different clinical forms might be related to the patient’s immune status, viral load and causative strain of the virus.

**Table 1:** The different forms documented in the literature for posterior herpetic uveitis in the immunocompetent

Necrotizing	PORN [15] Classic ARN
	Slow-type ARN [9]
	Multifocal posterior necrotizing retinitis [19]
	Focal retinitis in posterior pole [16]
Non Necrotizing	Vitritis, vasculitis and/or papillitis [8, 9]
	Panuveitis without distinct features [9]
	Occlusive vasculitis with early retinal neovascularization [17]

Whenever we think about the patterns of viral retinopathy there are always some baffling questions:

Why does the same virus give rise to different phenotypes while different viruses can cause the same pattern of retinitis? As the anterior herpetic uveitis is a true intraocular infection, why does the virus stay in the anterior segment the majority of the time? and what factors lead the virus to affect the posterior segment when it does happen? Why does the ARN pattern behave classically with characteristic peripheral patches of retinitis? How does the virus reach the other eye in BARN?

Our patient presented with the classical pattern of ARN in his left eye while the other eye did not show the usual anterior chamber or vitreous reaction, mimicking the behavior of viral retinitis in immunocompromised patients, and instead had posterior pole vasculitis with unusual pattern of optic neuropathy. The discrepancy in the clinical pictures between the two eyes posed a diagnostic challenge, especially that the patient revealed late the history of oral steroid use. The differential diagnosis of the striking optic disc hemorrhage in the right eye included Terson syndrome and hemorrhagic papilledema which compelled us to rule out cerebral vasculitis or any intracranial pathology as a cause of intracranial hemorrhage manifesting as Terson syndrome which was previously reported with ARN [20]. MRI and MRA brain were free. Therefore the only explanation for the right eye findings was severe vasculitis and inflammation involving the optic nerve head.

Extrapolating the findings of the first enucleated eye to confirm the herpetic etiology of ARN, Culbertson WW [3] reported that “the optic nerve was largely necrotic and heavily infiltrated with plasma cells.”

The right eye findings of our patient can be explained by a similar pathology described by Culbertson.

The conspicuous optic disc hemorrhage of the right eye is indicative of venous not arterial pathology caused by mechanical venous obstruction at the optic nerve head which is infiltrated by the virus particles and inflammatory cells squashing the vulnerable venous channels.

Such patho-mechanism was previously recognized as a rationale for the surgical optic nerve sheath decompression offered by Sergott et al. [21] for treatment of ARN- associated optic neuropathy.

57% of ARN patients have associated optic neuropathy [22]. It can happen before, after or concurrently with the retinal necrosis. Several mechanisms have been proposed to explain the optic neuropathy associated with ARN: (1) Intraneural vasculitis, (2) loculated exudates within the optic nerve sheath causing compressive ischemia, and (3) inflammation and necrosis due to direct invasion of the optic nerve with the virus particles [23].

The optic nerve in ARN may look normal in case of retro bulbar involvement, [24] it can show either hyperemic or pallid edema, [25]. It may be associated with nerve fiber layer hemorrhage or it may show pallor on presentation [10].

Sergott et al. [21] defined absolute and relative criteria for optic nerve involvement in ARN. The absolute criteria included: (1) an afferent pupillary defect, not consistent with the retinal findings; (2) poor correlation between retinal findings, visual acuity and visual fields; or (3) sudden deterioration of visual acuity to 20/100 or less without corresponding retinal changes within a 24 to 36 h interval.

Relative criteria were defined as: (1) optic disc edema and (2) enlarged optic nerves and surrounding perineural space demonstrated by CT scanning and or B-scan ultrasonography.

Optic neuropathy and retinal ischemia are believed to be responsible for the poor visual outcome in ARN not the retinal detachment [26]. The optic nerve involvement in our patient with multilayered retinal hemorrhage, unlike the previously described patterns, represents a novel phenotype of herpetic optic neuropathy.

The optic nerve was suggested to be the conduit through which the virus spreads to the brain and from the brain to the contra lateral eye via the contra lateral nerve [27].

Our patient presents circumstantial evidence that the spread of infection from the first eye to the second eye was along the optic nerve which was the only ocular structure to be involved in the second eye and if we have a look on the animal experiments to explain the transmission of the viral infection between both eyes we can get remarkable insights.

Oslon et al. [28] carried out experiments on rabbits in which HSV-1 was inoculated into the anterior chamber or vitreous cavity of one eye in normal rabbits and in rabbits with one optic nerve transected, and they concluded that HSV-1 can leave the inoculated eye by multiple routes depending on the site of virus inoculation, but that the virus reaches the retina of the contra lateral eye via the optic nerve only.

Whittum et al. [29] proposed upon another experiment on mice that HSV-1 presented into the anterior chamber elicits within the eye a local protective response, which is able in some manner to spare the ipsilateral retina from direct virus-mediated cytopathic effects. Whatever the nature of this local factor, it did not disseminate systemically to afford protection to the retina of the contra lateral eye.

Subsequently Azumi et al. conducted the same experiment of Whittum on T cell- and NK-cell depleted mice and they reported that the virus spread to both retinas, confirming a role for both cell types in the proposed protective process in the control of virus infection [30].

We believe that Whittum proposal can explain the usual observation that most viral infections involving the cornea and the anterior segment do no progress to involve the posterior segment. We advocate that failure of the development of that local protective mechanisms (due to the reported functional abnormalities in cellular immunity in ARN patients [12, 13] or the introduction of systemic or local steroids [31, 32] impaired the development of the proposed protective mechanisms. This results in spread of the virus to the posterior segment.

The peripheral patches of retinal opacification can not be explained only by the vascular occlusion but by the direct invasion of the virus to the retinal cells causing its destruction. Since these areas do not appear or behave the same way after healing as any healing retina after the usual arterial occlusion.

We may think that the PORN pattern could be ensued from only a different entry of the virus to the posterior segment circumventing the immune response of the anterior chamber and that phenotype can’t be explained by the systemic immunosuppression status of these patients alone that is why it is also reported in immunocompetent patients and the pathway of the virus in PORN follows the retrograde trans-synaptic transfer along the visual pathways from the optic nerve to the deep outer retinal layers then it spreads from the outer to the inner layers of the retina as the disease progresses, emerging as fulminant and multifocal retinitis with early involvement of the posterior pole sparring the anterior segment and vitreous cavity. The immunocompromised patients are more vulnerable to catch such opportunistic infection through any site of the body allowing the endogenous spread of the virus from the CNS to the eye along the optic nerve causing the usually reported PORN pattern in these patients. (When initially described by Forster et al. [7] 1990, PORN was associated with systemic cutaneous varicella zoster infection).

The absence of the significant vitreous reaction in typical PORN patterns can still be explained by the immunocompromised state of these patients. The overlapping atypical patterns of early posterior segment involvement and the presence of significant vitreous reaction [19].  Can be due to endogenous spread of the virus through the optic nerve in immunocompetent patients who can mount the regular inflammatory response.

So we can conclude that the PORN pattern may be caused by endogenous infection propagated through the optic nerve and the ARN pattern caused by exogenous herpetic infection or infection disseminated through the nasociliary nerve introducing the virus to the anterior segment and peripheral retina.

This variable routes of entry of the virus to the eye can explain the failure of Culbertson [33] to detect the viral particles in the optic nerve of his ARN case but the direct detection of the VZV by Greven et al. [34] in immunocompromised patient.

Margolis et al. [35] reported a unique case of a patient who was recovering from parasellar surgery and had completed a course of high dose systemic corticosteroids when she presented with a well-defined arcuate band of retinitis paralleling the course of a parafoveal nerve fibre bundle, he gave it a name of arcuate neuroretinitis. Later she developed peripheral retinitis and vasculitis. This patient may be representing the simultaneous occurrence of the 2 pathways with a focal infection of a limited number of retinal ganglion cells.

Corticosteroids have potent inhibitory effects particularly on cellular immunity. They are well known to increase the viral shedding and can even activate any latent viral infection. The literature is rich in reports documenting the development of serious herpetic uveitis after systemic or local intra- or periocular steroids without prior antiviral coverage [31, 32].

We believe that the erroneous administration of oral steroids for our patient was behind the rapid and aggressive progression of necrotizing retinitis in his first involved eye and the early and unusual involvement of the other eye.

## Conclusion

Herpetic etiology should be considered while managing different patterns of uveitis. Classical patterns of herpetic uveitis are easily recognized by clinical examination, but atypical patterns require a high index of suspicion and a low threshold for diagnostic tap.

Posterior viral uveitis is one of the most serious of infectious uveitides. It can deteriorate rapidly if not recognized early and treated promptly.

As a general rule in uveitis management, systemic or local steroids and steroid-sparing agents should not be prescribed before excluding and treating any possible infectious causes.

With the increasing reports of new and overlapping patterns and presentations, it is time to initiate an expert-led consensus to standardize a nomenclature system to define the different phenotypes of herpetic uveitis for clinical and research purposes and to review the possible pathogenesis of the previously described phenotype after the newly reported uncategorized patterns.

## Data Availability

The data used in that case report is available from the corresponding author on reasonable request.
